# Kaempferol blocks neutrophil extracellular traps formation and reduces tumour metastasis by inhibiting ROS‐PAD4 pathway

**DOI:** 10.1111/jcmm.15394

**Published:** 2020-05-19

**Authors:** Jie Zeng, Han Xu, Pei‐zhi Fan, Jing Xie, Jie He, Jie Yu, Xianwen Gu, Chao‐jie Zhang

**Affiliations:** ^1^ Department of Thyroid and Breast Surgery Hunan Provincial People’s Hospital Changsha China; ^2^ The First Affiliated Hospital Hunan Normal University Changsha China

**Keywords:** breast cancer, Kaempferol, metastasis, NETs, neutrophil

## Abstract

Kaempferol (kaem) is a dietary flavonoid found in a variety of fruits and vegetables. The inhibitory effects of kaem on primary tumour growth have been extensively investigated; however, its effects on tumour metastasis are largely unknown. In the present study, we found that kaem significantly suppresses both primary tumour growth and lung metastasis in mouse breast tumour model. Furthermore, decreased expression of citrullinated histone H3 (H3‐cit), a biomarker of neutrophil extracellular traps (NETs), had been founded in metastatic lung upon treated with kaem. The reduction of H3‐cit is not, however, due to the cytotoxicity of kaem on neutrophils since the frequency of CD11b^+^Ly6G^+^ neutrophils did not change in lung, tumour or blood in the presence of kaem. We then confirm the anti‐NETs effects of kaem in vitro by co‐culturing mouse neutrophils and kaem. Supplementing the neutrophils with GSK484, a potent NET inhibitor, totally abrogated the inhibitory effects of kaem on tumour metastasis while having little or no impact on primary tumour growth, indicating the specificity of kaem acting on NET formation and tumour metastasis. We also found that kaem suppressed ROS production in mouse bone‐marrow derived neutrophils. Supplementing with the ROS scavenger DPI abrogated kaem's effects on NET formation, suggesting the involvement of kaempferol in NADPH/ROS‐NETs signalling. Finally, we applied the kaem on NET‐deficient PAD4^‐/‐^ mice and found decreased primary tumour volume and weight but similar lung metastatic tumour with kaempferol treatment. Therefore, our findings reveal a novel mechanism of kaem in breast cancer development by targeting NETs induced tumour metastasis.

## 1 INTRODUCTION

Breast cancer is the most frequently diagnosed form of cancer and the second leading cause of cancer‐related death in women worldwide, with more than half a million women succumbing to the disease annually.^1^ Death, along with most of the complications associated with breast cancer, is due to metastatic growth in regional lymph nodes and in distant organs, including bone, lung, liver and brain.^2^ There are many ways to manage this leading cause of mortality, such as surgery, radiation treatment and chemotherapy. However, none of these is a panacea for such a resilient disease. The high risk of recurrence is a major clinical manifestation and represents the principal cause of breast cancer‐related death.^3^ Chemotherapy is also limited by a narrow therapeutic index, significant toxicities and acquired resistance.

Kaempferol (kaem), which is a widely distributed dietary flavonoid, mostly found in fruits and plants such as apples, strawberries, tomato, broccoli, green tea and ginkgo leaves.^4^, ^5^, ^6^, ^7^ Current studies have demonstrated the bioactivity of kaem, including its anti‐cancer, anti‐inflammation, antioxidant, antidiabetic, neuroprotective and cardioprotective abilities. With regard to its anti‐cancer activity, kaem regulates cancer cells through several different mechanisms, including but not limited to cell cycle arrest, promoting apoptosis and anti‐angiogenesis properties.^8^ However, the effects of kaem on tumour metastasis and cancer immunology remain unclear.

Neutrophils, which are a type of polymorphonuclear leukocyte, are well recognized as one of the major players during acute inflammation.^9^, ^10^ They are typically the first leukocytes to be recruited to an inflammatory site and are capable of eliminating pathogens via multiple mechanisms. Recent studies have revealed that the presence of a high neutrophil‐to‐lymphocyte ratio (NLR) has been associated with increased mortality in metastatic breast cancer,^10^ suggesting a role of neutrophil in tumour metastasis. Specifically, the metastasis‐supporting effects of neutrophil in breast cancer are related to the neutrophil extracellular traps (NETs).^11^ The effects of NETs in tumour metastasis are confirmed by many studies, but the underlining mechanisms are varied. Some researchers think that the DNA mesh can trap circulating cancer cells at the site of dissemination and promote metastasis,^12^ some think that NETs could activate toll‐like signalling and MAPK signalling, which could increase tumour cell survival,^13^ and other researchers think that NETs could increase local vascular permeability,^14^ and aid cancer cell to extravasate from the blood vessel more easily.

In this study, we found kaem significantly inhibited breast cancer cell metastasis in vivo. These anti‐metastatic effects were associated with inhibitory effects of kaem on neutrophil NET formation and dsDNA release. Treatment of the neutrophils with GSK484, a potent NETs inhibitor, totally abrogated the inhibitory effects by kaem, indicating the specificity of kaempferol acting on NETs. Because the formation of NETs highly relies on the ROS production by neutrophil, we confirmed that kaem could suppress neutrophil ROS release, which contributes to its inhibitory effect on NET formation. Consistent with these in vitro results, deletion of NETs in transgenic PAD4^−/−^ mice abrogated the anti‐metastasis effects of kaem in vivo. Therefore, our findings reveal a novel mechanism of kaempferol in breast cancer development by targeting NETs induced tumour metastasis.

## 2 MATERIALS AND METHODS

### 2.1 Drugs, reagents, antibodies and mice

Cancer cell line 4T‐1 was obtained from the American Type Tissue Culture Collection (ATCC). The cell line was grown in Roswell Park Memorial Institute 1640 medium (RPMI) (Thermo Scientific, MA, USA), supplemented with 10% foetal bovine serum (FBS) (GIBCO), 2 mmol/L glutamine (GIBCO) and 100 U/mL penicillin and streptomycin (GIBCO). The cell line was cultured at 37°C in a humidified chamber with 5% CO2. Kaempferol (Sigma‐Aldrich, USA) was dissolved in dimethyl sulphoxide (DMSO) to make a 10 mg/mL stock solution and stored at −20°C. The working dosage was freshly prepared in the basal medium with a final DMSO concentration of less than 0.1%. Anti‐MPO antibody (AF3667) was sourced from R&D, anti‐H3 citrulline (ab5103) and anti‐H3 (ab18521) from Abcam. All of the animals used in the experimental procedures were approved by the Institutional Animal Care and Use Committee and Institutional Review Board of Hunan Provincial People's Hospital (No. 2014‐07). All animals were kept under pathogen‐free conditions at room temperature (21 to 25°C), with 12 hours light/ dark exposure. Food and water were offered ad libitum. Bodyweight was measured twice weekly as an indicator of overall animal health. All efforts were made to ameliorate the suffering of the animals during euthanasia by overdosing with CO_2_ followed by cervical dislocation. Neutrophils were incubated with inhibitors 30 minutes before stimulation, as indicated. 20 μmol/L of the NADPH oxidase inhibitor, diphenyleneiodonium (DPI; Sigma‐Aldrich, 300 260), was used. Further, phorbol 12‐myristate 13‐acetate (PMA, Sigma‐Aldrich, P1585) was used at 10 nmol/L, NETs inhibitor GSK484 (Sigma‐Aldrich, SML1658) was used at 100 μmol/L, exosome inhibitor GW4869 (Sigma‐Aldrich, D1692) was used at 20 μmol/L, and the cells were incubated at 37°C. After 3 hours stimulation, cells were spun down and cell‐free supernatant was collected. Supernatants were stored at −20°C until used.

### 2.2 Cell proliferation

The exponentially growing cells (1 × 10^4^) were plated in media in 48‐well plates and treated with a series of concentrations of kaem dissolved in DMSO (final concentration ≤ 0.1%) after 24 hours of growth. Incubation was carried out at 37°C for 24 hours. Controls were formulated in DMSO vehicle at a concentration equal to that in drug‐treated cells. The media was removed, and plates were washed with phosphate‐buffered saline (PBS), air dried, stained with 0.3 mL methylene blue (2.5 g in 250 mL EtOH + 250 mL H2O), then left at room temperature (RT) for 2 hours. This was followed by washing with water, addition of 0.5 mL 1% Sarkosyl, and rotating at RT for 3 hours. 150 μL of this mixture was transferred to a 96‐well plate, and the OD at 595 nm was determined using a plate reader. Cell viability assay was performed with three independent experiments.

### 2.3 Wound‐healing assays

Confluent cells were cultured with 25 µmol/L kaem for 24 hours and then wounded with a linear scratch by a disposable 200 μL micropipette tip. Wounded monolayers were then washed four times with RPMI cell culture medium to remove cell debris, and incubated in 10% FBS RPMI medium, with or without kaem, for up to 24 hours. Cells migrated into the wound surface and the number of migrating cells were determined under an inverted microscope (Leica DMI 6000 B, IL, USA), by analysing five randomly chosen fields for each well. The percentage of inhibition was expressed using untreated wells at 100%. Three independent experiments were performed.

### 2.4 Matrigel invasion assay

Cells were treated with 25 µmol/L kaem for 24 hours, and then harvested and seeded in a Transwell chamber (Corning Inc, NY, USA) at 37°C. After stimulating cells with 10% foetal calf serum for 24 hours, non‐migratory cells on the upper surface were removed by a cotton swab. Invaded cells on the lower surface of the membrane were fixed with methanol and stained with crystal violet. The invaded cells were photographed (Leica DMI 6000 B) and quantified by manual counting. Ten randomly chosen fields were analysed for each group. Five independent experiments were performed.

### 2.5 Lung metastasis in breast cancer mouse model

Female BALB/C mice (Animal Center of Hunan province, China), between 7‐8 weeks of age and weighing 18‐22 g, were used for this experiment. 50 000 4T1 cells were cultured, prepared in matrigel (50 μL per mouse), and implanted into the 4th mammary fat pad of the anaesthetized mice, using intraperitoneal injection of Xylazin/Ketamine mix at a dose of 10 mg/kg and 100 mg/kg bodyweight, respectively. Bodyweight of each animal was recorded every 3rd day, until the end of the study in order to monitor well‐being of the animals. The animals were divided into vehicle group and kaem treated groups. The vehicle group (n = 8) received DMSO, and treatment group (n = 8 for each) received different doses of kaem. Treatments were administered twice per day by oral gavage, starting from day 15 of tumour implantation. The animals were killed at the end of week 6. Blood, lungs and tumour were collected for additional analysis. In another set of experiments, we set the end‐point when primary tumours attained the same size after different durations of time and then measured the lung metastatic nodules. The method of euthanasia for the animals used in this study was via administration of carbon dioxide.

### 2.6 Immunohistochemistry staining

After animals’ perfusion, the lungs were collected, kept in 3% paraformaldehyde and 3% sucrose solution, processed and paraffinized. Lung sections were stained for haematoxylin and eosin (H&E) to detect clear metastatic foci. Tissue sections were also stained for NETs markers such as MPO and citrullinated histone H3 (cit‐H3). In brief, the sections were de‐paraffinized, retrieved using 10 mmol/L citrate buffer at pH 6.0, and incubated at 95‐100°C for 10 minutes. The sections were then incubated with protein block (10% FBS in PBS) followed by incubation with the primary antibody diluted in PBS, and kept at 4°C overnight. Then, the sections were washed with PBS and incubated with fluorescent secondary antibody. For analysis, five randomly selected areas from each section were photographed at 40× magnification, using Leica SP2 confocal microscope (Leica, IL, USA), and the number of labelled and unlabelled cells per area was determined by two independent observers using ImageJ software (NIH).

### 2.7 Western blot analysis

Once proteins were extracted, they were quantified using Pierce BCA Protein Assay Kit (Thermo Scientific). The total cellular protein extracts were separated by SDS‐PAGE and transferred to polyvinyldifluoride (PVDF) membranes (Millipore, MA, USA). The membranes were blocked with 5% (w/v) non‐fat dry milk in TBST for 1 hour at room temperature, and then incubated overnight at 4°C with primary antibody. Blots were washed three times in TBST buffer, followed by incubation for 1 hour at room temperature with the corresponding HRP‐linked secondary antibodies. Specific proteins were visualized using enhanced chemiluminescence reagent (Thermo Scientific).

### 2.8 Flow cytometry analysis

Organs were collected (n = 8 from each group) for flow analysis. After extracting the tumour and lung tissues from mice, they were digested with collagenase II (1 mg/mL) and DNase (20 µg/mL) in RPMI media containing 5% FBS. Then, they were incubated at 37°C on a shaker for 1 hour and disseminated into single cells, filtered through a 40‐micron mesh, and spun at 500*g* for 15 minutes. The pellet was re‐suspended in fresh 1x PBS Mg/Ca (Hyclone, GE Healthcare Life Sciences, Logan, UT, USA), and supplemented with mouse LEAF blocker (Biolegend) for 15 minutes. Next, cells were labelled to detect CD45^+^/CD11b^+^/Ly6G^+^ neutrophils, CD45^+^/CD3^+^ T cells, CD45^+^/B220^+^ B cells, CD45^+^/CD3^‐^/NK1.1^+^ NK cells and CD45+/Ly6G‐/CD11b + monocytes. All antibodies were mouse specific and purchased from Biolegend. Flow cytometry samples were acquired using Accuri C6 flow cytometer (BD Biosciences, CA, USA) and analysed by BD Accuri C6 software.

### 2.9 SYTOX Green plate reader assay for NETosis analysis

Neutrophils were isolated from mouse bone marrow using EasySep Mouse Neutrophil Enrichment Kit (STEMCELL Technologies Inc., MA, USA). NETs were generated using the protocol described earlier.^15^ Briefly, Neutrophils were stimulated with 10 nmol/L PMA and incubated on a 25 mm flat tissue culture dish for 4 hours. After 4 hours, the media was gently aspirated and discarded, and the bottom layer was collected by pipetting 15 mL cold PBS (no Ca^2+^ and Mg^2+^). The suspensions were then centrifuged at 450 g for 10 minutes at 4°C to collect the cell‐free NET‐rich supernatant. The supernatant was divided into 1.5 mL tubes and spun for at 18 000 g 10 minutes at 4°C. Supernatant was then discarded and the pellet was resuspended in 100 µL of PBS. Concentration of the sample ranged between 140 and 180 ng/µL.

### 2.10 Citrullination assay

HEK‐293T cells were transiently transfected with HA‐tagged PAD4 plasmid using Lipofectamine 3000 reagent (Thermo Fisher). After 24 hours, cells were lysed in a solution comprising 50 mmol/L Tris‐HCl (pH 7.4), 1.5 mmol/L MgCl2, 5% glycerol, 150 mmol/L NaCl,0.4% NP40 and 1 mmol/L DTT with protease inhibitor cocktail (Roche, Basel, Switzerland). Lysates were pre‐incubated for 30 minutes at 4°C with vehicle or 100 µmol/L kaem. Citrullination reactions were performed for 30 minutes at 37°C in the presence of 2 mmol/L calcium. Extracts were loaded on to gels; proteins were separated by SDS‐PAGE and transferred to PVDF membranes. Citrullinated proteins were then chemically modified following manufacturer's instructions (Sigma, MO, USA, 17‐347B) and detected using anti‐modified citrulline antibody (Abcam, Cambridge, UK, ab6464) and HA antibody (Novus Biologicals, CO, USA, NB600‐362).

### 2.11 Statistical analysis

Quantitative data were expressed as standard error of the mean (± SEM) and analysed through Student *t* test or ANOVA (depending on the number of comparative groups), followed by Bonferroni test for normal distribution. Those groups with non‐normal distribution were analysed using the Mann‐Whitney test or Kruskal‐Wallis test. Statistical significant differences were considered at *P*‐value < 0.05 using GraphPad Prism 6 software.

## 3 RESULTS

### 3.1 Kaem inhibits breast tumour metastasis in mice

**FIGURE 1:**
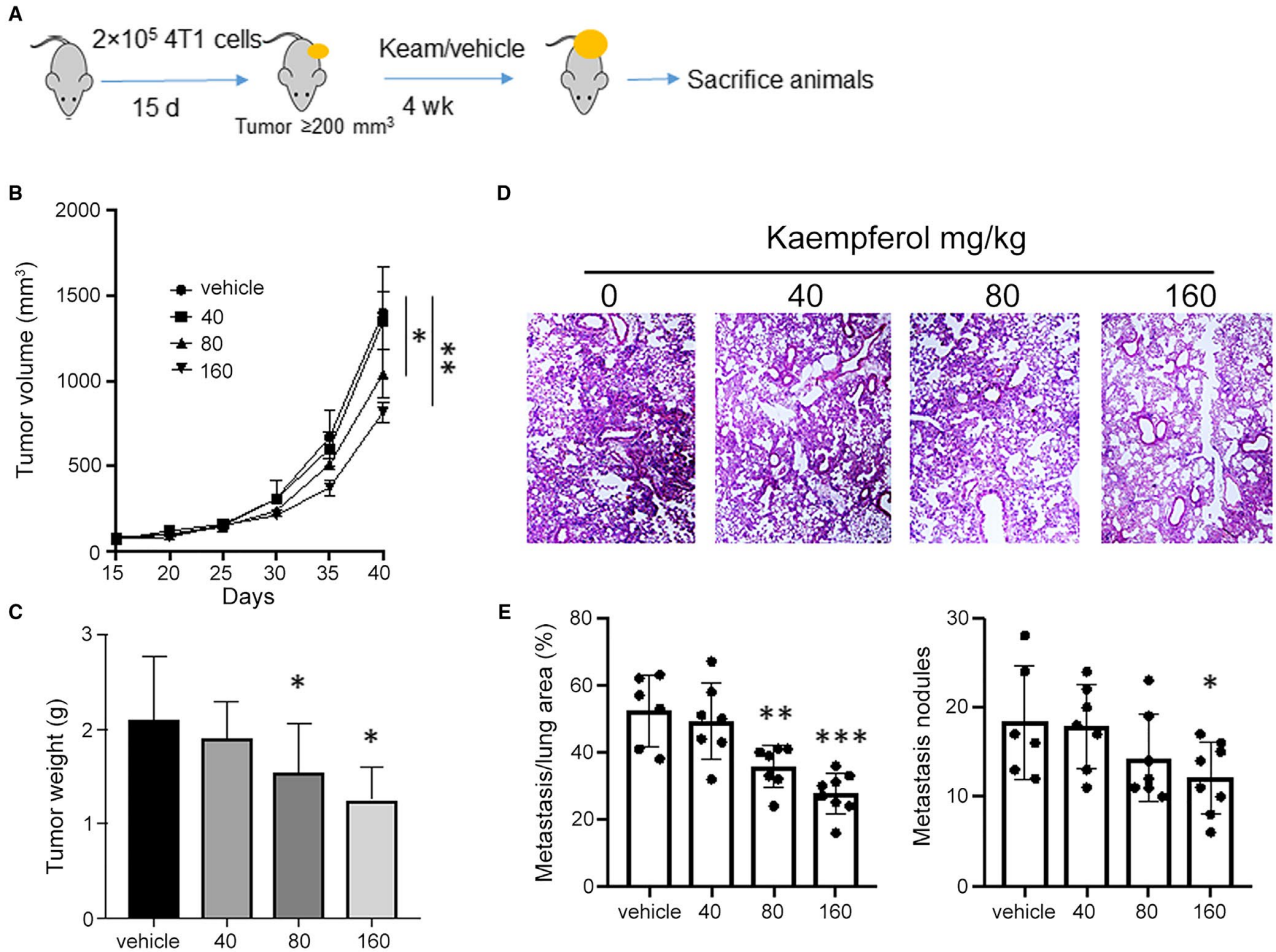
Kaempferol inhibits tumour growth and metastasis in vivo*.* 50 000 4T1 cells were implanted into the 4th mammary fat pad of the mice, followed by administration with vehicle, or 40 mg/kg, 80 mg/kg, 160 mg/kg kaem via oral gavage, once per day. (A) Schematic overview of regimen for examination of primary tumour development and metastatic tumour formation in the lung utilizing Kaempferol. (B) Measurement of tumour volume. (C) Measurement of tumour weight. (D) H&E staining for metastatic lung. (E) The percentage of metastatic tumour area in whole lung sections. Data presented as mean ± SEM of three independent experiments. *P < 0.05, compared with control group; **P < 0.01, compared with control; ***P < 0.001, compared with control

We first examined the effect of kaem on the primary tumour growth and metastasis of 4T1 cells in BALB/C mice using ectopic implantation of cancer cells in the mammary fat pad (Figure 1A). This tumour models allowed us determine the formation of both primary and secondary metastatic growth in breast tumours. For the primary tumour growth, dose dependent effects can be seen between 40 and 160 mg/kg kaem. Upon treating with 160 mg/kg kaem, the primary tumour volume decreased from 1500 mm^3^ to 800 mm^3^ (Figure 1B‐C) and no significant bodyweight loss can be seen. We also assessed the primary tumour and metastasis in the lung by H&E staining (Figure 1D, Figure S1). In the primary tumour sections treated with only the vehicle, the tumour cells were packed tightly, distributed diffusely and interspersed with plentiful stroma. The tumour cells showed large nuclei with a clear nucleolus. However, after treatment with kaem, the tumours exhibited significantly different histological properties. A decrease in the percentage of the nuclei, indicated by blue spots, was observed in all kaem‐treated groups (40 mg/kg, 80 mg/kg and 160 mg/kg) when compared with the vehicle group. Meanwhile, nuclear shrinkage and fragmentation were also observed, indicating typical apoptotic characteristics induced by kaem. Moreover, in the 160 mg/kg treated groups, the necrosis area increased significantly, and only a small portion of tumour cells remained. These observations suggested the dose‐dependent antitumour effect of kaem. Additionally, the metastatic burden in the lung showed a striking decrease from 52% (percentage of lung area occupied by metastasis) to 31%, upon treatment with kaem at the dosage of 160 mg/kg (Figure 1D‐E). As metastasis involves the spread of cancer cells from the primary tumour, in order to rule out the possibility of primary tumour growth inhibition by kaem, we set the end‐point when primary tumours reached the same size after varying durations, and then measured the lung metastatic nodules. Kaem treatment still significantly inhibited tumour metastasis (data not shown). To investigate the mechanisms by which kaem inhibits tumour growth and metastasis, we tested its effects on tumour proliferation and invasion. Consistent with previous studies, kaem significantly inhibited the viability of breast cancer cells by more than 50% at 100 µmol/L. However, there were no effects on wound healing and transwell tests. Those observations indicated that although kaem could suppress 4T1 cell proliferation, its effect on cell invasion or metastasis is limited (Figure S2A‐C).

### 3.2 Kaem inhibits neutrophil extracellular trap formation in vivo and in vitro

**FIGURE 2:**
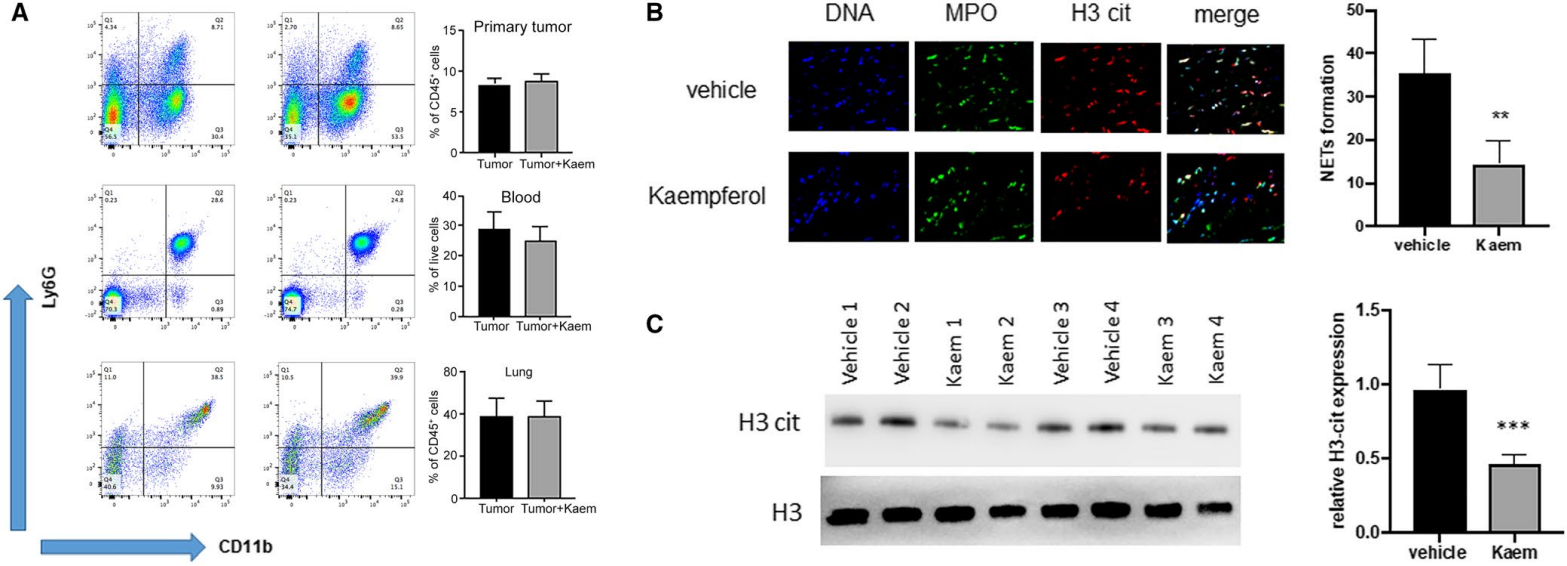
Kaempferol inhibits NET formation in vivo. (A) Flow cytometric analysis of the neutrophil proportions in primary tumour, blood and whole lung tumour‐bearing mice and kaem (160 mg/kg, oral gavage) treated mice. (B) Confocal staining of DNA, H3‐cit and MPO in metastatic lungs. DNA (blue), citrullinated histone H3 (Cit‐H3, red), MPO (green). (C) Western blot for H3‐cit expression in metastatic lungs. Data presented as mean ± SEM of three independent experiments. *P < 0.05, compared with control group; **P < 0.01, compared with control; ***P < 0.001, compared with control

To address the discrepancy in the in vitro and in vivo anti‐metastatic effects demonstrated by kaem, we decided to measure the neutrophils in the animal model, especially since neutrophils have recently emerged as important players in breast tumour microenvironments, particularly in metastasis. We collected samples of primary tumour, blood and metastatic lung from tumorous mice with and without kaem treatment (Figure 2A). Analysis revealed clear neutrophil populations in primary tumours, blood and metastatic lungs, but no significant difference in the neutrophil numbers between control mice and kaem‐treated mice. These results suggest that the neutrophil survival or infiltration is not impaired by kaem. We also measured other immune cells in the metastatic lungs, such as T cells, NK cells, B cells and monocytes. Our data showed that kaem did not affect infiltration of those immune cells in metastatic lungs (Figure S3). However, the expression of DNA and citrullinated histone H3 (H3 cit), a biomarker of neutrophil extracellular trap formation, was suppressed by half in MPO^+^ neutrophils in metastatic lungs upon kaem treatment (Figure 2B and C), indicating that the kaem may contribute to regulation of neutrophil NET formation.

**FIGURE 3:**
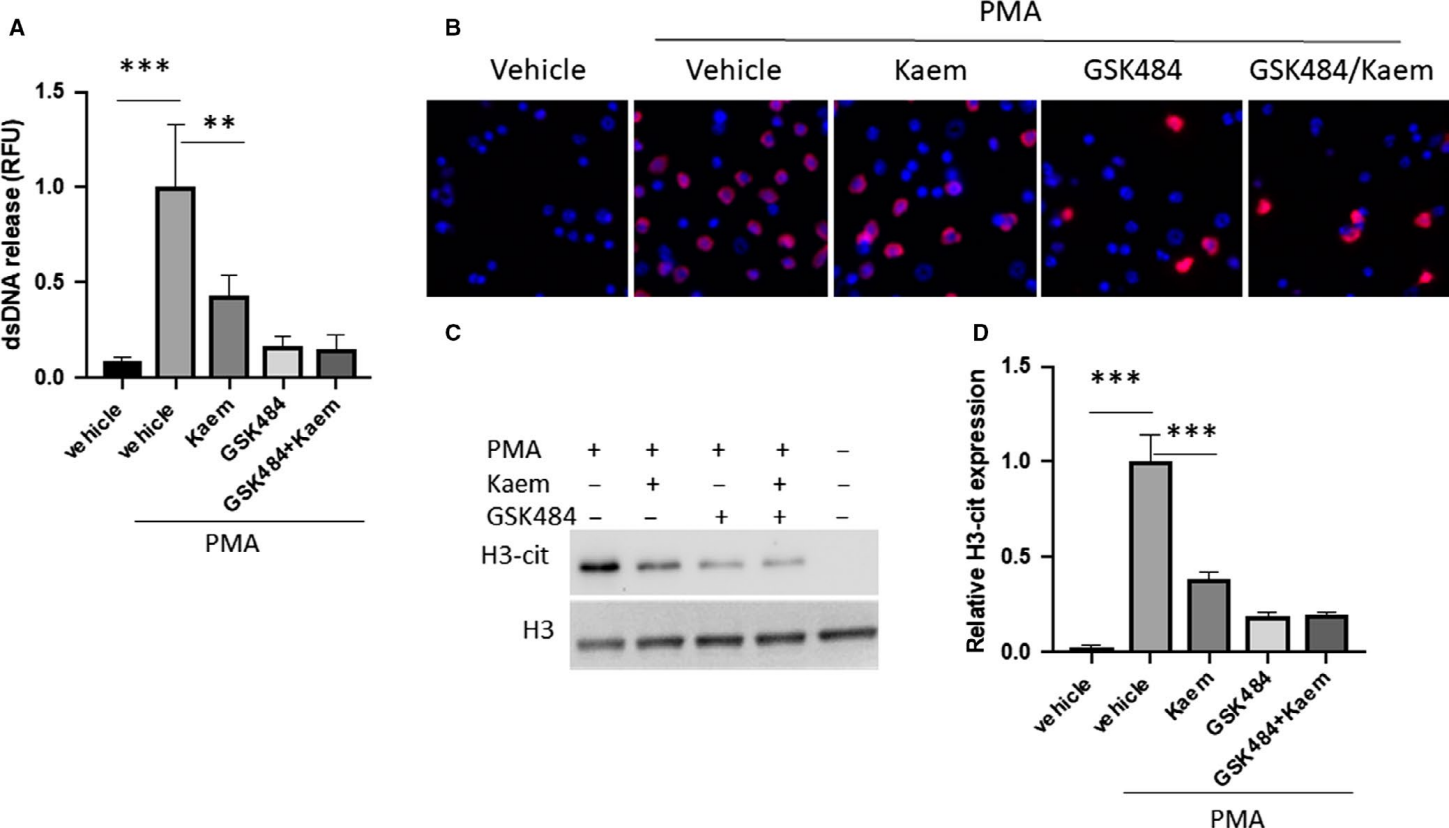
Kaempferol inhibits NET formation in vitro*.* (A) Primary mouse neutrophils stimulated with PMA (10 nmol/L) to induce dsDNA release. Supplementing the stimulated neutrophils with kaem (100 μmol/L), GSK484 (100 μmol/L), or a combination of both significantly inhibited NET formation. (B) Confocal image showed decreased DNA release. DAPI (blue), MPO (red). (C) H3‐cit expression by Western blot. (D) Quantification data for western blot. Data presented as mean ± SEM of three independent experiments. *P < 0.05, compared with control group; **P < 0.01, compared with control; ***P < 0.001, compared with control

Since kaem treatment showed NETs inhibitory effect in metastatic lungs, which might contribute to its anti‐metastasis effect, our next question was whether the effect is directly driven by kaem, or mediated by other cells or micro‐environmental factors. Therefore, we isolated fresh neutrophils from mouse bone marrow and stimulated them with phorbol 12‐myristate 13‐acetate (PMA) to induce NET formation. Supplementing the mouse primary neutrophils with kaem could inhibit double‐strand DNA release, a hallmark of the formation of NETs. The reversible PAD4 inhibitor, GSK484, which blocks the citrullination of PAD4 targeted proteins in neutrophils and inhibits the formation of NETs, also showed significant reduction of dsDNA release (Figure 3A). Moreover, kaem could not further reduce dsDNA release with GSK484 pre‐treated neutrophils (Figure 3A). In the functional kinetic analysis, in which the PAD4 was overexpressed in HEK293 cells, the expression of citrullinated proteins was significantly inhibited by the kaem treatment (Figure S4), suggesting that kaem may inhibit NET formation through a PAD4‐dependent signalling pathway. We further confirmed our hypothesis by performing immunofluorescence assay and Western blot (Figure 3B‐D).

### 3.3 Neutrophil extracellular traps promote breast cancer cell migration

**FIGURE 4:**
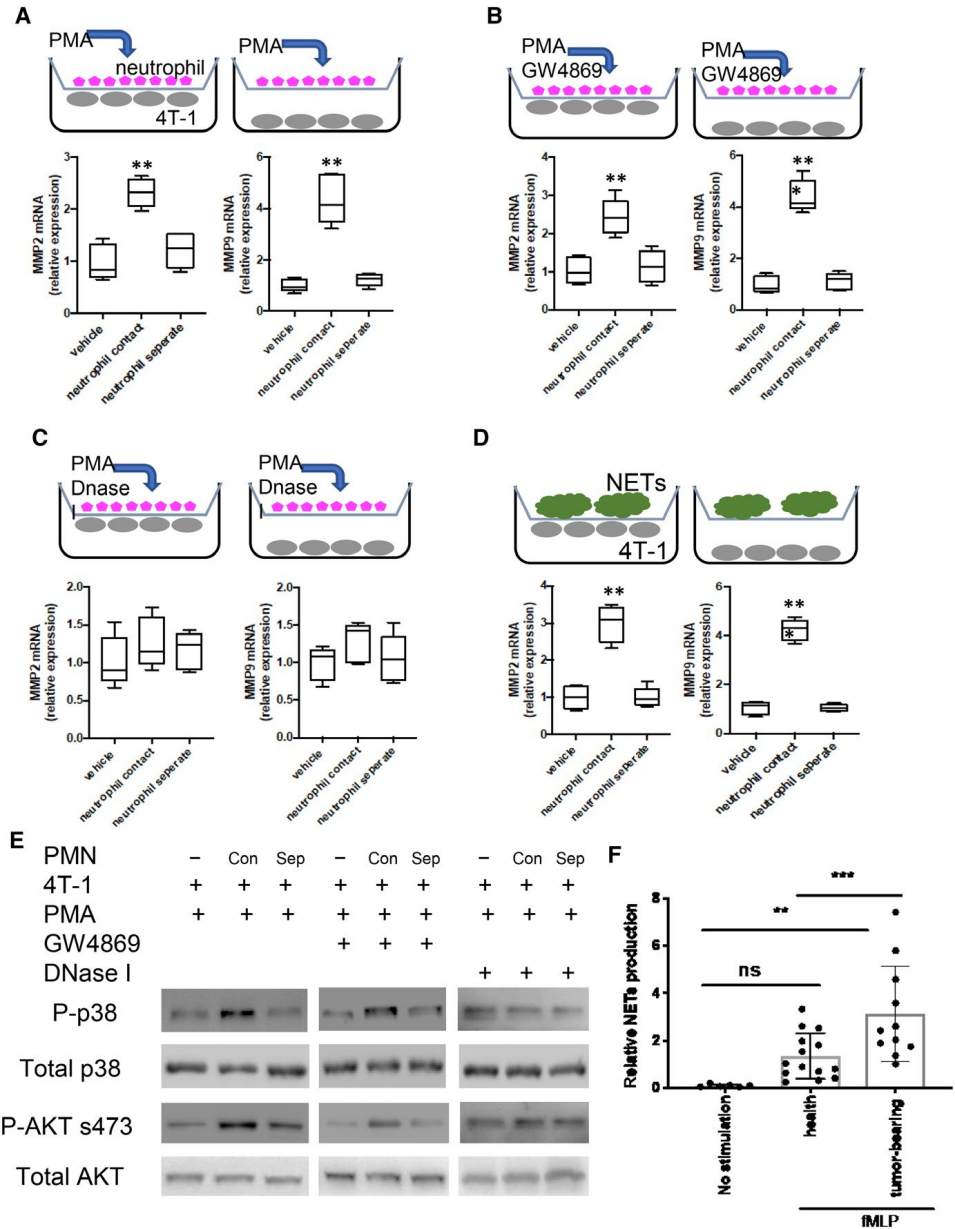
NETs promote breast cancer cell survival and metastasis. Primary mouse neutrophils and 4T‐1 cells were co‐cultured at opposite sides of transwell permeable supports (referred to as contact model). Neutrophils were grown on transwell permeable supports and 4T‐1 cell were grown on the bottom of a separate well, the gap between the two cell layers being 0.8 mm (referred as separate model). MMP2 and MMP9 mRNA levels were measured by 10 nmol/L PMA treated for 2 h (A), pre‐treated with 20 µmol/L exosome inhibitor GW4869 for 1 h, and then added 10 nmol/L PMA for additional 2 h (B), followed by pre‐treatment with 1000 U DNAse I for 1 h and addition of 10 nmol/L PMA for another 2 h (C), and 4T‐1 cells co‐cultured with isolated NETs (300 mg/dL of protein) (D). (E) NETs production of neutrophils from health and breast cancer patients. (F) Western blot of p38 and AKT pathway for samples from 5A‐C. Data presented as mean ± SEM of three independent experiments. *P < 0.05, compared with control group; **P < 0.01, compared with control; ***P < 0.001, compared with control

Although recent studies indicate that neutrophils or NETs promote tumour metastasis, our understanding of these mechanisms is still limited. Here, we tested the effects of NETs on tumour cell migration in vitro by using co‐culture models with neutrophil and breast cancer cell lines 4T1. We chose two different co‐culture models, contact‐model and separate‐model (Figure 4A‐D). Enhancement of metastatic potency of breast cancer cells by neutrophils was demonstrated by increased MMP‐2 and MMP‐9 mRNA levels, as well as p‐p38 and p‐AKT protein levels only in the contact model. Pre‐treatment of the neutrophils with GW4869, an inhibitor of exosome biogenesis/release, or with glucocorticoids, a neutrophil degranulation inhibitor still showed a significant stimulatory effect on tumour metastasis (Figure 4B). However, pre‐treatment of the neutrophils with DNase I suppressed the effects of the neutrophils, suggesting that neutrophil extracellular traps (NETs) play a central role in breast cancer cell metastasis (Figure 4C), which is consistent with previous studies.^11^ To further confirm our observation, we isolated NETs from mouse bone marrow neutrophil and co‐cultured these with 4T1 cell line, which perfectly mimics the neutrophil co‐culture phenotype (Figure 4D). In addition, we performed a transwell assay using the isolated NETs‐4T1 cell co‐culture model. It showed that NETs dramatically increased the migration of 4T1 cell (Figure S5). Inhibitory effects of DNase I on p‐p38 and p‐AKT signalling were also confirmed by Western blot, indicating that the NETs could promote p38 and AKT pathway signalling for cell survival and metastasis (Figure 4E). Interestingly, a significant increase in NETs production in response to fMLP was detected in the neutrophils of patients who developed invasive breast cancer, when compared with age/gender‐matched healthy volunteers (Figure 4F).

### 3.4 Kaem inhibits neutrophil extracellular traps formation through ROS signalling

**FIGURE 5:**
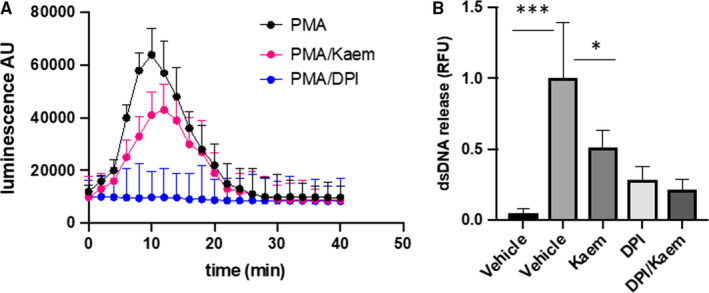
Inhibition of NET formation by Kaempferol depends on ROS signalling. (A) Primary mouse neutrophils stimulated with PMA at 10 nmol/L to induce ROS production. Pre‐treatment of the stimulated neutrophils with kaem (100 μmol/L) or DPI (20 μmol/L) for 30 min at 37°C significantly inhibited ROS production. (B) dsRNA released by PMA‐stimulated neutrophils is inhibited by kaem or DPI, under the same condition. Data presented as mean ± SEM of three independent experiments. *P < 0.05, compared with control group; **P < 0.01, compared with control; ***P < 0.001, compared with control

NADPH oxidase derived reactive oxygen species (ROS) is indispensable for the process of NET formation.^16^ Since kaem is a polyphenol antioxidant compound,^17^ it is worthwhile to test its effect on ROS production in neutrophils and NET formation. By using the classical PMA‐induced ROS production model, we found that kaem could inhibit ROS production and dsDNA release in active neutrophils (Figure 5A). However, kaem could not further lower the dsDNA release if the cells were pre‐treated with Diphenyleneiodonium (DPI), an NADPH inhibitor (Figure 5B), suggesting that the inhibitory effect of kaem on NET formation is mediated by ROS inhibition.

### 3.5 Inhibition of NET formation in vivo fails to affect primary tumour growth, but abrogates the anti‐metastatic effect by kaem

**FIGURE 6:**
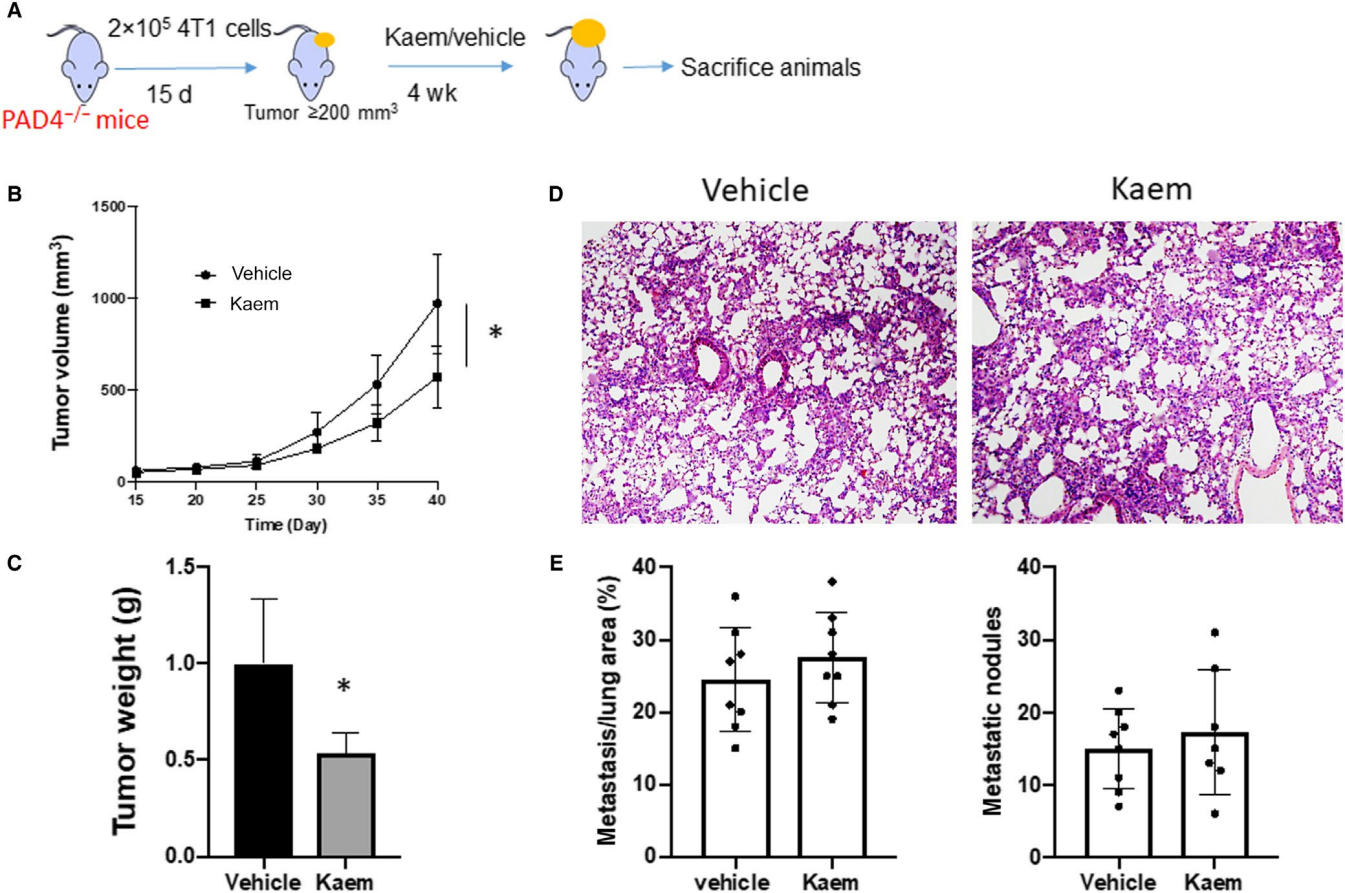
NET formation in vivo abrogates Kaempferol's inhibitory ability on tumour metastasis. (A) Schematic overview of regimen for examination of primary tumour development and metastatic tumour formation in the lung in PAD4 KO mice with Kaempferol treatment (160 mg/kg, oral gavage). (B) Measurement of primary tumour volume. (C) Measurement of primary tumour weight. (D) The percentage of metastatic tumour area in whole lung sections, H&E staining. (E) Quantification of H&E staining. Data presented as mean ± SEM of three independent experiments. *P < 0.05, compared with control group; **P < 0.01, compared with control; ***P < 0.001, compared with control

To verify the anti‐NETs effect of kaem in vivo, we use peptidyl arginine deiminase type IV (PAD4)‐deficient mice^18^ (Figure 6A). Compared to vehicle‐treated 4T1 tumour bearing PAD4^−/−^ mice, kaem treatment showed 50% reduction of primary tumour volume (Figure 6B), and 50% reduction of tumour weight (Figure 6C). However, there is no significant difference of the metastatic tumour in the lung between vehicle and kaem treated mice (Figure 6D‐E). These results suggested that kaem could inhibit both primary tumour growth and tumour metastasis by distinct mechanisms. The possible mechanism through which kaem inhibits tumour metastasis is mainly through the ROS‐PAD4 signalling.

## 4 DISCUSSION

Among the many cell types within the tumour microenvironment, immune cells such as lymphocytes, macrophages and neutrophils play a prominent role in tumour development and progression. Neutrophils, however, have only recently emerged as important players, particularly in metastasis.^19^ As highlighted in other recent studies, neutrophils can influence tumour metastasis via their ability to produce and release a wide variety of proteins into the extracellular environment. However, in our co‐culture model, we did not see any significant reduction of metastatic ability in mouse breast cancer cell lines if neutrophils and tumour cells were cultured separately in two chambers, indicating the influence between cells is limited by distance parameters. We also tested the role of exosomes in our co‐culture model, because as a newly discovered cell‐cell communication mechanism, neutrophil‐derived exosome can modulate cell migration, cell differentiation and other aspects of cell‐to‐cell communication. However, we still could not see significant reduction of metastatic ability in mouse breast cancer cell lines after GW4869 treatment. We do see a dramatic reduction by using NETs inhibitor DNase I treatment, which means NETs could promote cancer cell metastasis. This observation was also confirmed using co‐cultured purified NETs and cancer cell lines. The stimulatory effects of NETs in tumour metastasis have been confirmed by many studies, but the underlining mechanisms are varied. Some researchers think that the DNA mesh can trap circulating cancer cells at the site of dissemination and promote metastasis,^12^ some think that NETs could activate toll‐like signalling and MAPK signalling, which could increase tumour cell survival,^13^ while some other researchers think that NETs could increase local vascular permeability,^14^ and aid cancer cell to extravasate across blood vessels more easily. Due to the limitations of our co‐culture model, we could not get the possibility of the NETs effects on circulation cancer cells and vascular permeability, but we confirmed that NETs could activate MAPK signalling and MMP signalling.

**FIGURE 7:**
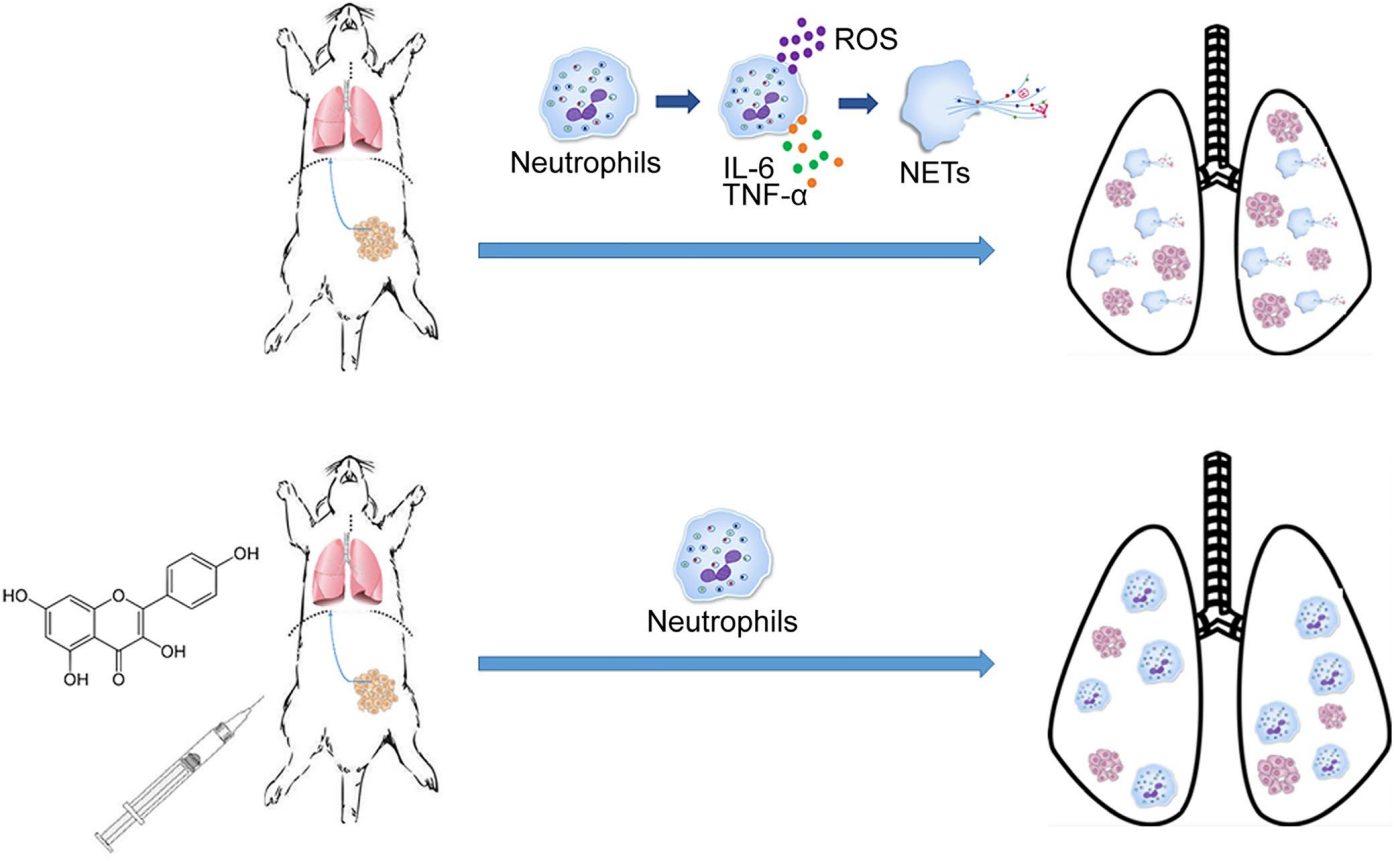
An overview of the mechanism of action of kaempferol in tumour metastasis

Although targeting NETs to prevent metastasis has been proposed by many papers and showed promising effects, the clinical or pre‐clinical medicine is still limited. Some newly discovered technology, such as DNase I‐coated nanoparticles,^11^ and newly discovered targets, such as PI3K‐gamma,^20^ propose a promising therapeutic strategy to block NET formation in vivo. However, their safety and efficiency need to be evaluated in the long run. In our study, we found kaem works well against breast tumour metastasis. Many studies focus on the anti‐proliferation or promote apoptosis effects of kaem on tumour cells. Here, we explore the anti‐metastatic effects of this popular flavonoid. In vitro results demonstrated that kaem inhibited NETs release in the isolated neutrophils with a significant reduction of citrullinated histone H3, a hallmark of NET formation. We also showed that kaem cannot further reduce NET formation with PAD4 blocker pre‐treatment, suggesting that kaem may inhibit NET formation through a PAD4‐dependent signalling pathway. This assumption was confirmed by PAD4’s functional kinetic analysis (measuring citrullination directly) and validated in PAD4‐deficient mice. However, we still cannot rule out the possibility of kaem reducing NET formation through other downstream, such as Syk, Src kinases or PI3K IAδ,^21^ which needs further investigation. Therefore, there is great future potential for kaem to work along or in combination with other reagents in order to treat metastatic tumours (Figure 7).

## CONFLICT OF INTEREST

All authors read and approved the final version of the manuscript, and the authors declare that they have no competing interests.

## AUTHOR CONTRIBUTIONS

Jie Zeng and Chao‐jie Zhang contributed to conception and design of the work; Jie Zeng, Han Xu, Pei‐zhi Fan, Jing Xie and Xianwen Gu contributed to data acquisition; Jie Zeng, Han Xu, Pei‐zhi Fan, Jing Xie, Jie He, Jie Yu and Xianwen Gu contributed to data analysis; Jie Zeng and Chao‐jie Zhang contributed to draft the work. All authors contributed important intellectual content for the overall work and take responsibility for the honesty and accuracy of the present study.

## ETHICAL APPROVAL

The study was approved by the ethics committee of Hunan Provincial People's Hospital (No. 2014‐07). All patients provided written informed consent before taking part in the study.

## Supporting information

Fig S1

Fig S2

Fig S3

Fig S4

Fig S5

## Data Availability

The data that support the findings of this study are available from the corresponding author upon reasonable request.
