# Supplementary figures and images for: Kaempferol blocks neutrophil extracellular traps formation and reduces tumour metastasis by inhibiting ROS‐PAD4 pathway
Source: J Cell Mol Med. 2020 May 19;24(13):7590–9. doi: 10.1111/jcmm.15394 (PMC7339206; doi:10.1111/jcmm.15394)

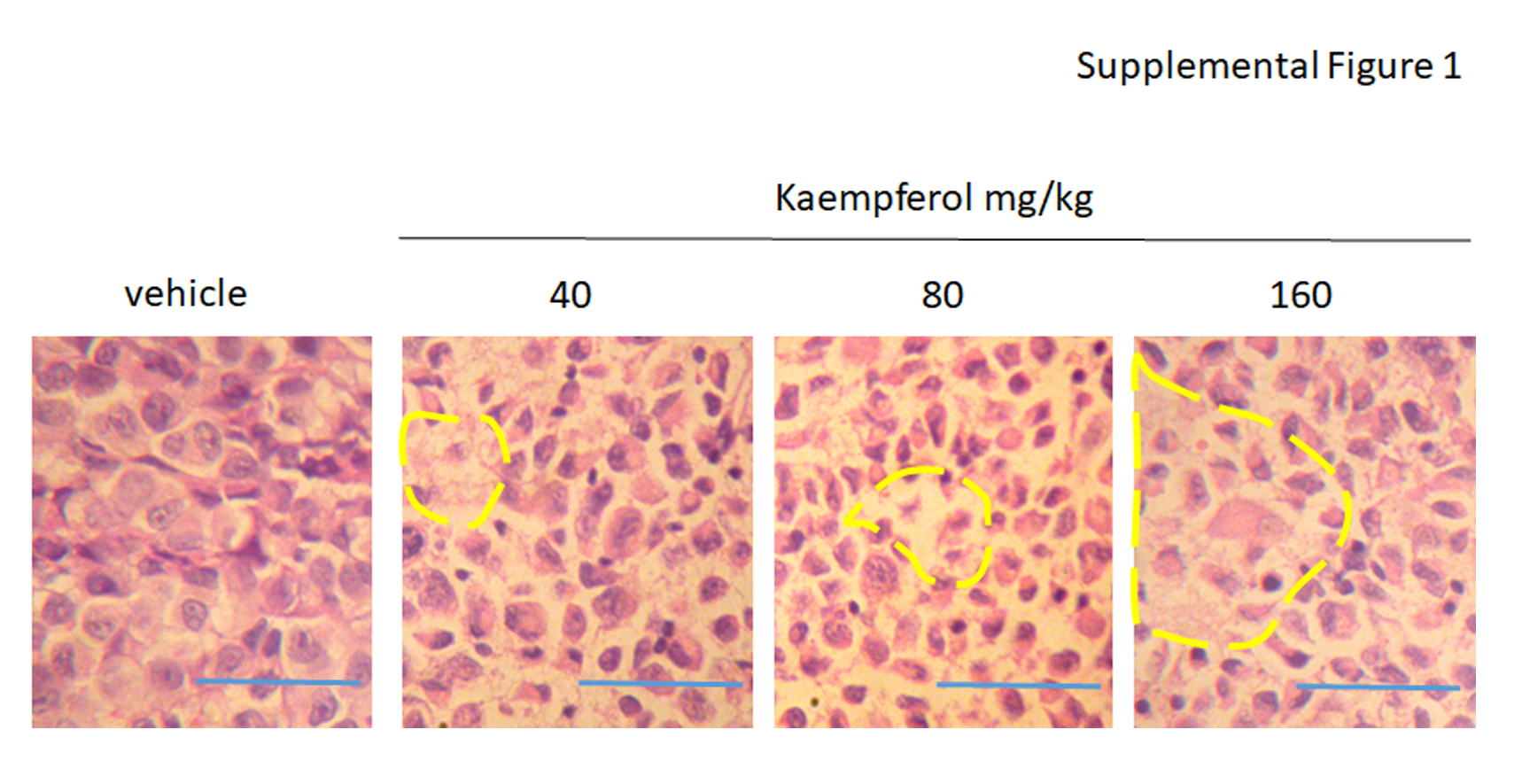

Supplement: Supplementary file 1 — Fig S1 [file JCMM-24-7590-s001.tif]

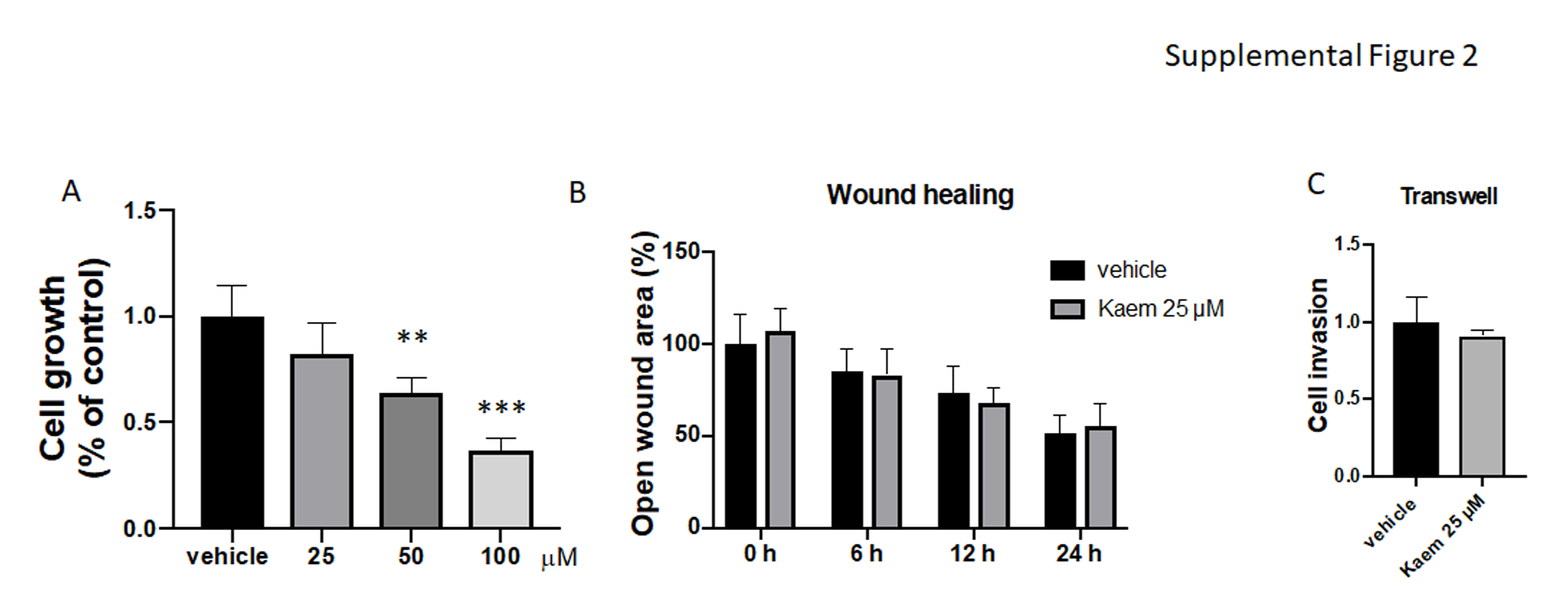

Supplement: Supplementary file 2 — Fig S2 [file JCMM-24-7590-s002.tif]

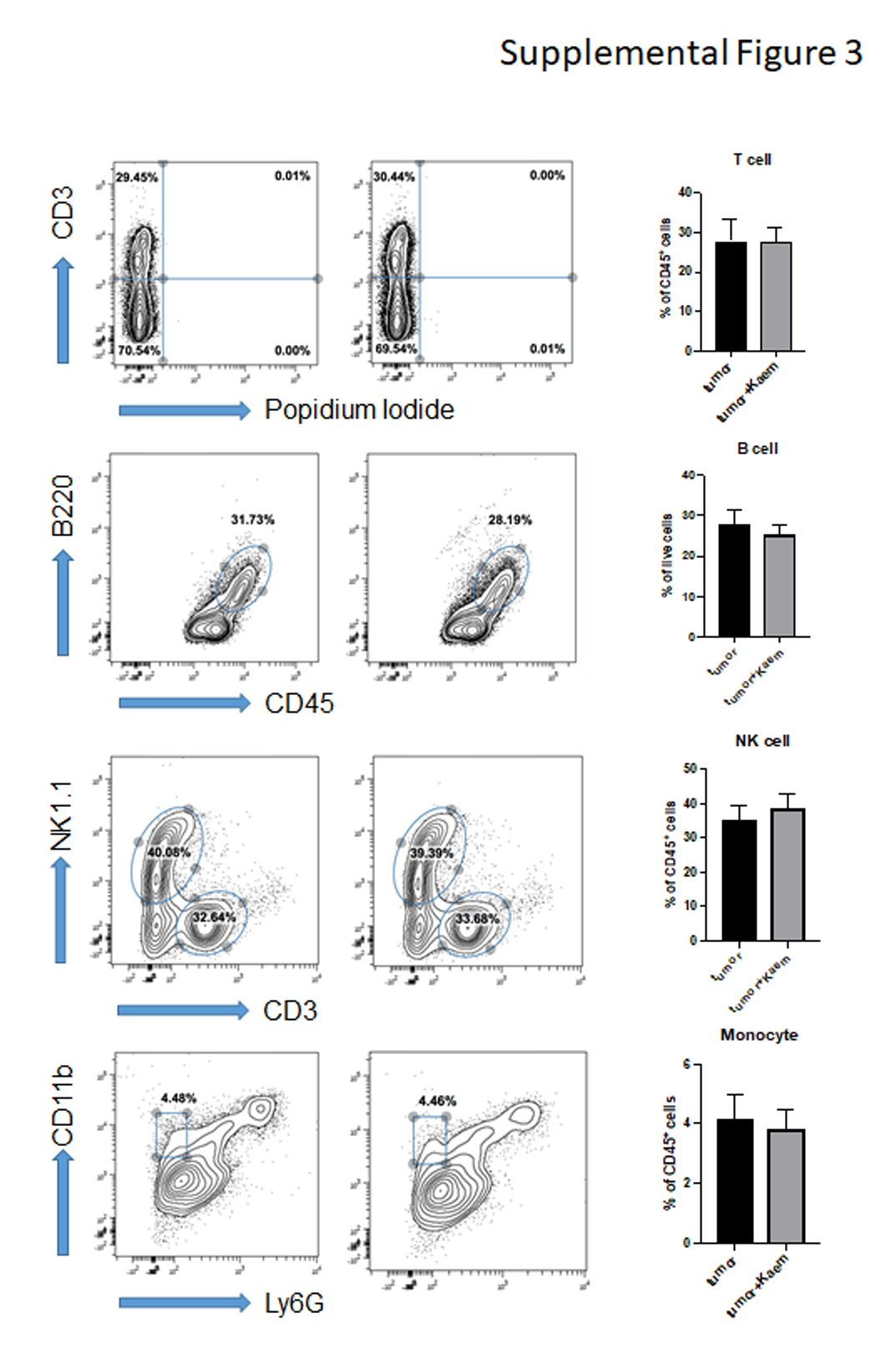

Supplement: Supplementary file 3 — Fig S3 [file JCMM-24-7590-s003.tif]

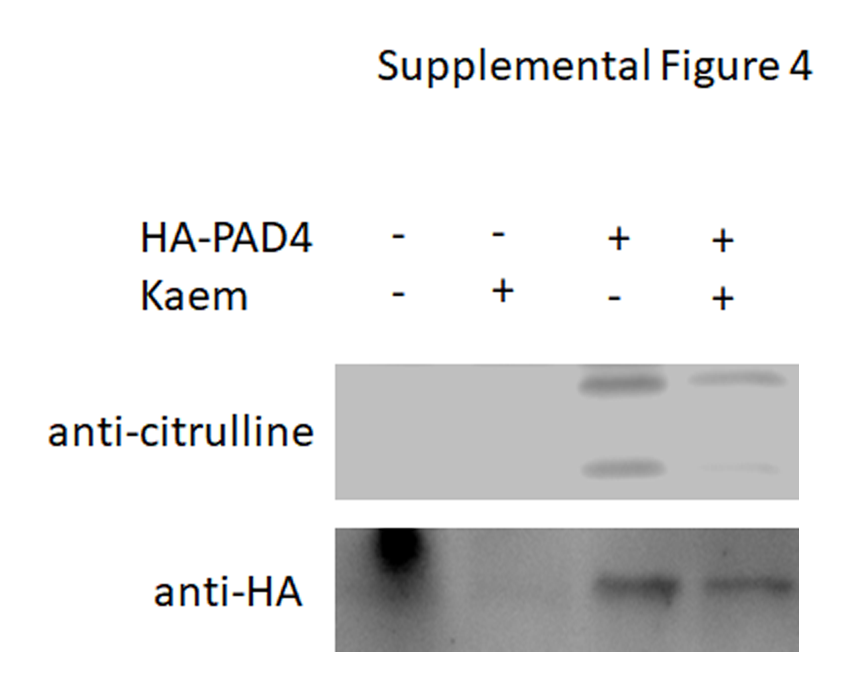

Supplement: Supplementary file 4 — Fig S4 [file JCMM-24-7590-s004.tif]

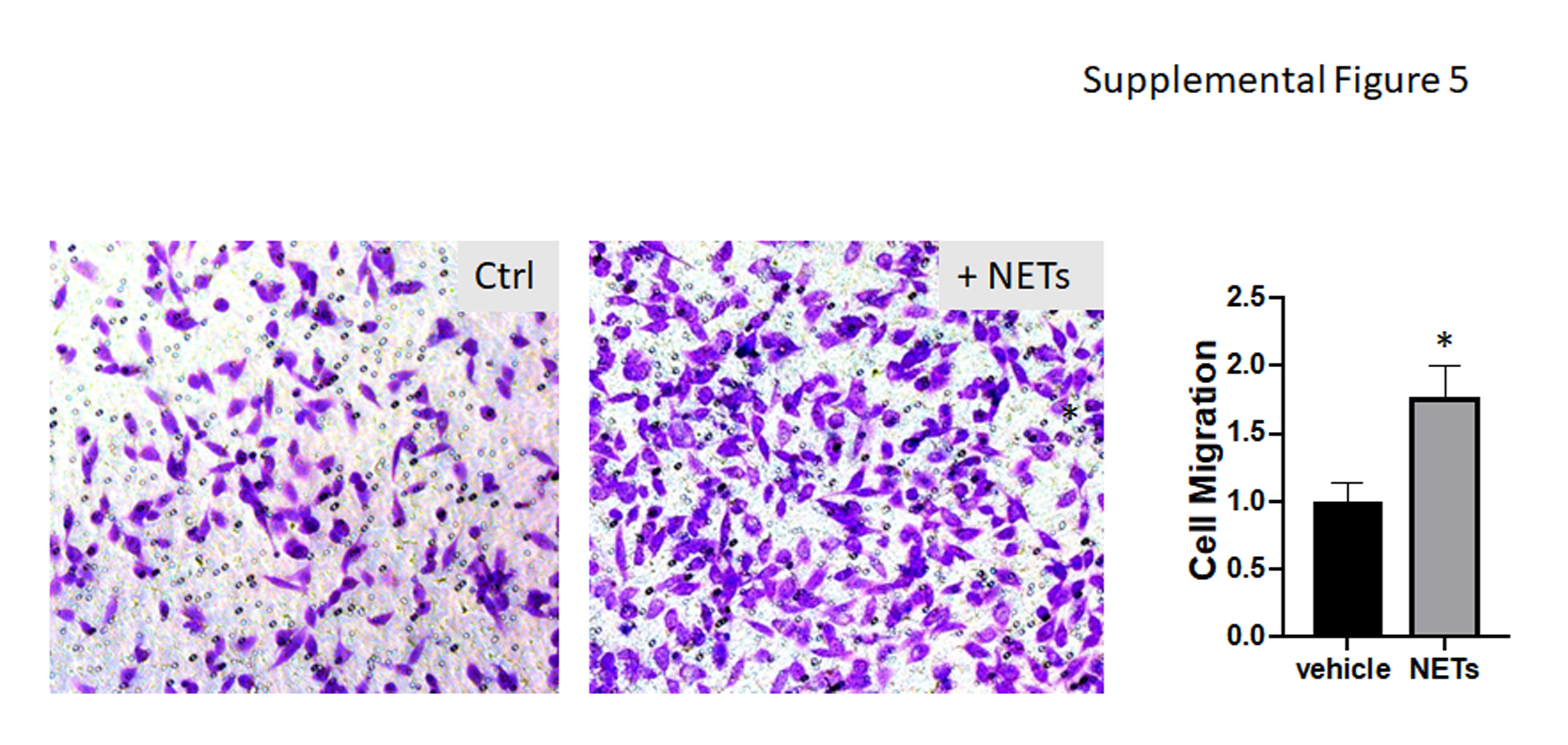

Supplement: Supplementary file 5 — Fig S5 [file JCMM-24-7590-s005.tif]
